# Software Code Smell Prediction Model Using Shannon, Rényi and Tsallis Entropies

**DOI:** 10.3390/e20050372

**Published:** 2018-05-17

**Authors:** Aakanshi Gupta, Bharti Suri, Vijay Kumar, Sanjay Misra, Tomas Blažauskas, Robertas Damaševičius

**Affiliations:** 1 Department of Computer Science and Engineering, Amity School of Engineering and Technology, New Delhi 110061, India; 2 University School of Information, Communication and Technology, Guru Gobind Singh Indraprastha University, New Delhi 110078, India; 3 Department of Mathematics, Amity School of Engineering and Technology, New Delhi 110061, India; 4 Center of Information and Communication Technology/Engineering (ICT/ICE) Research, New Building of Covenant University Center for Research Innovation and Development (CUCRID), Covenant University, Ota 112231, Nigeria; 5 Department of Computer Engineering, Atilim University, Incek 06836, Turkey; 6 Department of Software Engineering, Kaunas University of Technology, Kaunas 44249, Lithuania

**Keywords:** software design defects, software quality, code smell, entropy, statistical model, regression

## Abstract

The current era demands high quality software in a limited time period to achieve new goals and heights. To meet user requirements, the source codes undergo frequent modifications which can generate the bad smells in software that deteriorate the quality and reliability of software. Source code of the open source software is easily accessible by any developer, thus frequently modifiable. In this paper, we have proposed a mathematical model to predict the bad smells using the concept of entropy as defined by the Information Theory. Open-source software Apache Abdera is taken into consideration for calculating the bad smells. Bad smells are collected using a detection tool from sub components of the Apache Abdera project, and different measures of entropy (Shannon, Rényi and Tsallis entropy). By applying non-linear regression techniques, the bad smells that can arise in the future versions of software are predicted based on the observed bad smells and entropy measures. The proposed model has been validated using goodness of fit parameters (prediction error, bias, variation, and Root Mean Squared Prediction Error (RMSPE)). The values of model performance statistics ($R^{2}$, adjusted $R^{2}$, Mean Square Error (MSE) and standard error) also justify the proposed model. We have compared the results of the prediction model with the observed results on real data. The results of the model might be helpful for software development industries and future researchers.

## 1. Introduction

Object-oriented software systems (OOSS) are prone to continuous design changes. When any open source software (OSS) is developed, its source code is available for modification or enhancement by anyone. With frequent changes in the source code, the software becomes complex over a period of time [1,2]. These design changes and complexity in the software results in the introduction of code smells and at times they may cause the failure of the software.

Code smells are bad programming practices. They affect the software quality as well as maintenance cost of the software (software maintenance cost is around 60–80% of total software cost [3,4]). International Organization for Standardization (ISO) standard 8402-1986 elucidated software quality as “the totality of features and characteristics of a product or service that bears its ability to satisfy stated or implied needs” [5]. Code smells comply a negative impact on the software structural quality. Software structure quality reflects on the inner structure of the software and its source code. An important attribute of the software quality is the software reliability which is also affected by bad smells [6], as the bad smells can caused the failure of a software. Bad smells could be considered as the symptoms which indicate poor design quality of source code [7,8] or test code [9], and make a software hard to maintain and shorten its life cycle [10]. On the other hand, if code smells are detected early enough, it allows to reduce software testing costs and ensures higher software reliability.

Bad smells give a more scientific evaluation on where and how to refactor a software module. It is introduced during the software development due to lack of time or through a lack of a developer’s experience. With the maintenance cost of software on the rise, there is an increasing need for measuring the code smells at an early stage of the software development life cycle. It becomes a priority for software developers to manage and measure the complexity of the software code [11], as complex software code leads to high maintenance cost.

**Figure 1:**
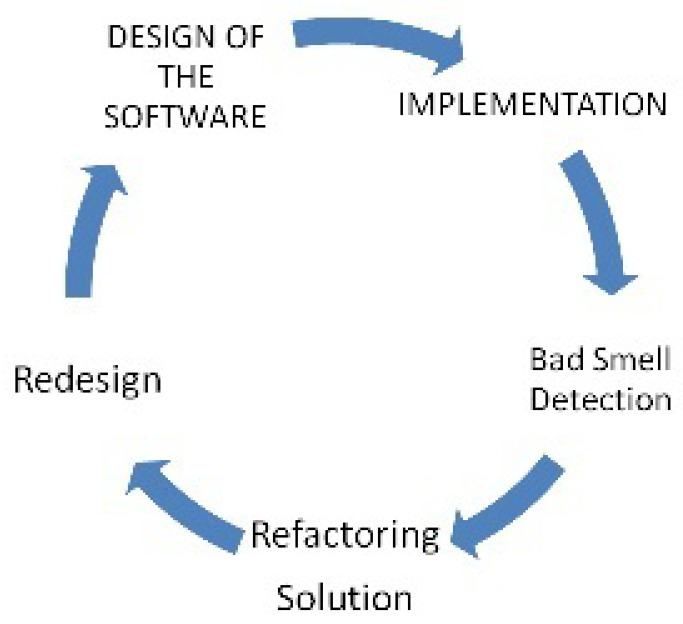
Common software life cycle with respect to bad smell prediction after implementation.

**Figure 2:**
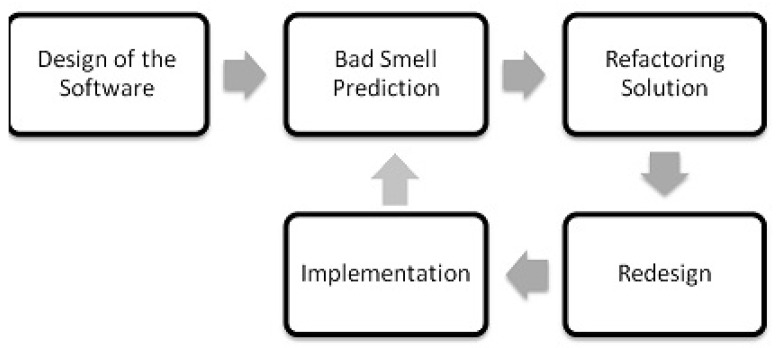
Software life cycle with prior bad smell detection.

Figure 1 shows the general life cycle of the software development in which bad smells are predicted after implementation of the code. In this model the re-factoring solutions [12] and the redesigning of the software are provided after bad smell detection. In Figure 2, bad smells can be predicted and removed before implementation process. It will reduce the development life cycle and increase the efficiency of the process.

Previous studies on bad smells and design errors offered many detection techniques [13], including expert-based approach [14] and logical prediction of bad smells using machine learning techniques [12]. In an existing software, a bad smell can be detected simply by using a tool and actions can be taken accordingly. However, if we want to predict the smells before being introduced in the software, we primarily base our confidence on an appropriate mathematical model. In this context, we consider a code smell estimation model that can be used to build relationships between code smells and the different versions of the software. These models can be either statistical models (such as a regression model) or logical models. The proposed approach suggests a statistical model. This model estimates probabilistic future behavior of the system based on past statistical information. This also helps to estimate uncertainties in observational data and in calculation based on observational data.

As per our knowledge, there is no research paper available in which any statistical model has been proposed till now to predict the bad smells. Though there are logical and structural models that exist to predict and hence reduce the number of code smells. Code smells present in the several versions of Apache Abdera software (1.1.3, The Apache Software Foundation, Forest Hill, MD, USA) are taken into consideration after data preprocessing. The major features of this study that make it unique and add to an already existing large pool of studies are:

- Calculated different measures of entropy namely, Shannon’s entropy, Rényi entropy and Tsallis entropy, for the bad smells on real data of Apache Abdera versions.
- Applied the non-linear regression on the computed entropy.
- Compared the predicted code smells with the observed code smells.
- Goodness of fit criteria and statistical performance metrics have been calculated to validate the proposed model.
- Calculated $R^{2}$ value to justify the model.

The rest of the paper is organized as follows. Related work of the code smell and information theory is described in Section 2, the information-theoretic approach is presented in Section 3 and Section 4 elaborates the experiment design and bad smell prediction modeling. Section 5 describes data collection and preprocessing, and Section 6 demonstrates the result and discussion of the proposed model with critical analysis and observations. Section 7 gives its application and limitations and Section 8 concludes the paper.

## 2. Related Work and Motivation

Software developers develop software to fulfill the needs of the end users. The quality of software is influenced by technical, human, environmental, and organizational practices [15]. Lack of quality management and assurance in IT industry leads to lower reliability of software delivered to end-users. Lots of techniques have been proposed for bad smell detection and software quality assurance. The prime motivation of this research was to develop a statistical model for predicting the bad smells and justify the validity of the model with the goodness of fit and statistical parameters.

Commonly, data from existing software source code versions or their testing data are used to develop software fault (or reliability) prediction models, which are then applied to predict future occurrences software defects or faults [16]. Researchers are also working hard to predict the bad smells in the software and the impact of bad smells on the maintenance. The bad smell term was coined Beck and Fowler [17] in the book “Refactoring: Improving the structure of existing code”. Mantyla et al. [18] introduced a new smell: “Dead code” that is never executed. They categorized the smells into six categories and analyzed the detection of code smells subjectively. Tufano et al. [7,19] empirically assessed about the reasons of bad smells occurred in the software and the survivability of the bad smells. They concluded their study over the change history of 200 open source projects. Chatzigeorgiou et al. [20] examined the evolution of four bad smells (long method, god class, state checking and feature envy) throughout successive release of two open-source systems. They stated that mostly the bad smells persist up to the latest analyzed release accumulated as the project matured. A variety of bad smell detection techniques such as binary logistic regression, clustering, genetic algorithm, and relation association rule mining have been tabulated in the literature [13].

For example, Zhang et al. [6,21] described quality assurance in code development by using code smells. Emden et al. [22] developed a Java code smell detector/tool and applied the tool in a case study. Moha et al. [23,24] implemented a tool (DECOR) in a domain specific language to detect the bad smells. Fontana et al. [25,26] examined that different tools mostly gives a distinct result, which is why the illustration of the threshold values and magnitude is arduous across tools. Dexun et al. [27] introduced a new concept of weight based distance metrics (Jaccard distance) for feature envy bad smell detection. The approach is applied for Jfreechart open source system and achieved high accuracy with low time complexity. Liu et al. [28] discovered a resolution sequence of bad smells and validated its effect on two nontrivial applications. Palomba et al. [29] presented the approach using change history information and detected five (Parallel inheritance, Divergent change, Shotgun surgery, Blob and Feature envy) bad smells. Hassaine et al. [30] proposed a systematic parallel between artificial immune system (AIS) for bad smell detection. They tested two systems (Gantt project and Xercess) for three (Blob, Functional decomposition, Spaghetti code) smells. Czibula et al. [31] identified faulty software entities such as classes, methods using relational association rule in object oriented systems. They compared the outcomes with other conventional computation techniques and prove the potential of their approach. Kessentini et al. [32,33,34,35,36,37] stated that due to code smells, the cost of maintenance has increased. They have also described that code smells can be prioritized on the basis of risk. Yamashita et al. [38,39,40,41] analyzed that maintenance cost also gets affected due to the interaction between the smells. Khomh et al. [42] identified the code smells, which affect the maintenance efforts and relationship of change proneness. Code clone, a type of code smell has been studied the most [6]. A survey on the behavior of the code clone and its applications have been described in the literature [43]. Holschuh [44] presented a report for the industry use that is based on the defect prediction of Java language depending on the code smell metrics. There is one logical prediction of bad smells that has been proposed by Maneerat et al. [12] using machine learning algorithms. In this model, seven data sets were considered with 27 design metrics and seven bad smells. Authors have also used the statistical analysis for the significance of the prediction. Taba et al. [45] proposed antipattern based metrics and bug prediction model to improve the accuracy of bug prediction, while Codabux et al. [46] related code smells to the number of micro and nano-patterns in source code. Zhu et al. [47] presented a software reliability model for Non-homogeneous Poisson process related to software fault dependency and imperfect fault removal. They considered two types of faults (dependent and independent) according to fault dependency. Amarjeet et al. [48] proposed a Harmony Search Based algorithm for software remodularization for object oriented systems. They have compared the proposed algorithm with other algorithms, in terms of Modularization Quality (MQ), Non Extreme Distribution (NED), authoritativeness, and searching efficiency and achieved the Harmony search based algorithm has better results to improve the quality of remodularization of software systems. Bansal [49] analyzed the change prone classes in the software systems using the Hybridized Search based algorithmic model and machine learning based models in predicting change prone classes of software using g-mean and accuracy.

However, the imbalanced distribution of software faults in source code leads to poor prediction power of machine learning techniques applied to predict source code defects such as bad smells [50]. Hassan [51] proposed the information theory concept to measure the amount of randomness or entropy of the distribution to quantify the code complexity as a result of code changes. Singh et al. [52] presented a mathematical model using entropy for bug prediction. Chaturvedi et al. [53] proposed a model to predict the bugs based on the current year complexity of code changes/entropy. Key difference between the proposed research work and the existing research papers has been summarized in Table 1.

## 3. Information Theoretic Approach

The information theory, a mathematical concept of communication that deals with assessing and defining the amount of information contained in a message, is measured as the amount of entropy/randomness of the distribution. The term entropy, denoted as *S*, was proposed by Shannon [54]. It is a vital branch of information theory which plays an important role in studying the code changes. It is an approach that is based on the probability concept that emphasizes the measurement of “randomness” related information. Entropy is applied in various domains, such as pattern detection, statistical inference, natural language processing, thermal physics, and quantum computing. The Shannon entropy *S* is defined as:

(1) $$S=-\sum_{i=1}^{n}\left(P_{i}\log_{2}P_{i}\right)\;\;where\;\;P_{i}>=0,\;\sum_{i=1}^{n}\left(P_{i}\right)=1$$

here $P_{i}$ is the probability of occurrence of an event and the value of *i* varies from 1 to *n*, and *n* is the number of files.

Entropy will be the maximum when for distribution *P*, all the files have the same probability of changes $\left(P_{i}=1/n;\;\forall i=1,2,\ldots ,n\right)$. On the other hand, if for a *P* distribution file, *k* has a probability of code changes, i.e., ($P_{i}=1$ and ∀i≠k) $P_{i}=0$, the entropy will be the minimum. From the definition of entropy we can state that if the changes are in every file then the entropy will be maximum and it will be minimum if the changes are in the single file.

A generalization of Shannon entropy in a systematic way has been characterized and developed by Rényi [55] as follows:

(2) $$R=\frac{1}{1-\alpha }\log\left(\sum_{i=1}^{n}P_{i}^{\alpha }\right)\;\;where\;\;\alpha \neq 1,\alpha >0$$

Tsallis [56] proposed another generalization of Shannon entropy [57,58] defined as:

(3) $$T=\frac{1}{\alpha -1}\left(1-\sum_{i=1}^{n}P_{i}^{\alpha }\right)\;\;where\;\;\alpha \neq 1,\alpha >0$$

Rényi and Tsallis entropy reduces to Shannon entropy when α →1. For Rényi [55] and Tsallis entropy [56], any value of α can be taken except 1 and must be α >0. To study the variation in entropy, five values of α parameter has taken into consideration, that is 0.1, 0.3, 0.5, 0.7 and 0.9.

Entropy helps in studying the process of code change. Due to the changes in the code, the code becomes complex and may produce the code smells. The process of code change refers to the study of code patterns. Feature enhancement, new feature addition and bug fixing cause these code changes or modifications. The frequent changes in the code may also degrade the overall quality, reliability and sustainability of the software system and introduce the code smells in the source code. To measure the effect of code changes in the software instead of simply counting the number of changes, entropy quantifies the pattern of changes. These changes are calculated for a particular duration like ranging from hours to years/decades. The frequency of updates in software leads to the release of different versions of software.

For example, consider a software system which in total has 13 bad smells in four classes and three versions. These bad smells in classes with respect to versions are shown in Table 2. If C1, C2, C3, C4 are the classes and Version 1, Version 2 and Version 3 are three versions. In Version 1, class C1, C2, C4 have one bad smell each and C3 has two bad smells. Table 2 depicts the number of bad smells in three versions with respect to classes. Total bad smells in Version 1 are five. Thus, the probability of occurring bad smells for Version 1 is 1/5=0.2. Similarly, we can calculate the probabilities of all the available versions with respect to each class. Based upon these probabilities the Shannon entropy [57,58], Rényi entropy [55], and Tsallis entropy [56] are determined.

## 4. Experiment Design and Bad Smell Prediction Modeling

This paper aims to achieve two objectives. The first objective aims to develop a mathematical model for predicting the code smells. The second aims to verify the result of the prediction model with the help of the statistical parameter $R^{2}$.

### 4.1. Evaluation of Entropy

We have used a matrix representation between the classes and seven versions of the Apache Abdera software which calculates the entropy for the mathematical model in this study. For this, we’ve used a tool for code smell detection in each class of the software. The tool, Robusta (version 1.6.9, https://marketplace.eclipse.org/content/robusta-eclipse-plugin) calculates the occurrence of bad smells in the class of each version termed as probability. Using these probabilities, Shannon, Rényi and Tsallis entropy have been calculated with the help of Equations (1)–(3) respectively for each version of the software. For the Rényi and Tsallis entropies, five values of α are considered as 0.1, 0.3, 0.5, 0.7 and 0.9.

These entropy values represent the independent variables in this paper. The variable to be predicted for the bad smell prediction is called the dependent variable. With the help of non-linear regression, we have developed a mathematical model for predicting the bad smells using entropy measures.

### 4.2. Bad Smell Prediction Modeling

In the nonlinear regression model, observational data has been modeled by a function in which model parameters are combined non-linearly and depend on one or more independent variables. In this work, the independent variable entropy has been measured for various versions of Apache Abdera software. Once the entropy is measured, the bad smells are predicted using nonlinear regression. Entropy is the independent variable that is represented by *X* and predicted bad smell is presented as *Y*, the dependent variable. Thus, the following nonlinear regression model is proposed:

(4) $$\begin{array}{c} Y=A+B\times E+C\times E\times E \end{array}$$

here

- *E* = (*S*, *R*(0.1), *R*(0.3), *R*(0.5), *R*(0.7), *R*(0.9), *T*(0.1), *T*(0.3), *T*(0.5), *T*(0.7), *T*(0.9)) are entropies,
- *Y* are predicted bad smells,
- *A*, *B*, *C* are the regression coefficients.

A variable *E* is the entropy which takes different value that is Shannon entropy [57,58], Rényi entropy [55], Tsallis entropy [56]. For Rényi and Tsallis entropy, five values of parameter α are considered, namely, 0.1, 0.3, 0.5, 0.7 and 0.9.

## 5. Data Collection and Preprossessing

### 5.1. Software Project Used as a Case Study

The data consisting of six bad smells is extracted for seven official releases of Apache Abdera project. This is performed using detection tool Robusta. Apache Abdera is a large corporate strength open-source system. The Abdera was developed initially by IBM and donated to the Apache Abdera software foundation. These smells are given with their description in Table 3. The bad smells are then determined using a detection tool from the classes of Apache Abdera. They are then analyzed with respect to their classes and an excel worksheet is populated with the values of bad smells. Shannon, Rényi and Tsallis entropies have been calculated for each version of the Apache Abdera. IBM SPSS Statistics (version 2015, IBM Corporation, Armonk, NY, USA) regression analysis is used to predict the bad smells for the coming release.

We have used Robusta (version 1.6.9, National Taipei University of Technology (Taipei Tech), Taipei, Taiwan), a plug-in tool for Eclipse to identify the classes that had the bad smells. The source code of Abdera has been complied on this plug-in. Table 4 shows the compiled result of the detected code smells in the classes of Apache Abdera software.

### 5.2. Assessment of Shannon, Rényi and Tsallis Entropy

The collected data have been used to calculate the probability of all available seven versions of Abdera software as discussed in Section 3. Using probabilities, the value of entropies: Shannon, Rényi and Tsallis have been calculated. Equations (1)–(3) have been used respectively to calculate the entropies as discussed in Section 3.

Five values of α example 0.1, 0.3, 0.5, 0.7, 0.9 are considered for Rényi and Tsallis entropy as discussed in Section 3. Table 5 depicts the entropy values for each version calculated using Equations (1)–(3). Section 3 also describes the example for calculating the entropies with the derived data. Here *S* stands for Shannon entropy, *R*(0.1) stands for Rényi entropy with α 0.1 value and *T*(0.1) stands for Tsallis entropy with α values. We observed that Shannon entropy values lie between 1 to 4 and when the value of α increases, the values of Rényi and Tsallis entropy decrease.

### 5.3. Model Construction

**Figure 3:**
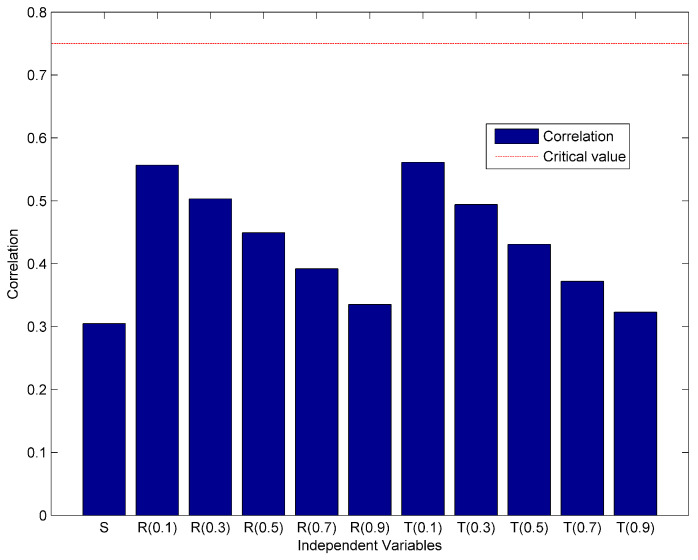
The results of correlation analysis assuming linear model.

In order to construct and validate the prediction model, we follow the methodology and recommendations suggested by Stockl et al. [59]. First, we perform the correlation analysis to check if the model inputs and outputs are linearly related. The null hypothesis is that the population correlation coefficient equals 0. For a sample size of seven, the Pearson‘s *r* value for α = 0.95 significance is 0.75 [60]. The results of correlation analysis are presented in Figure 3. We can see that all correlation values are below critical *r* value, suggesting that the model is not a linear one.

**Figure 4:**
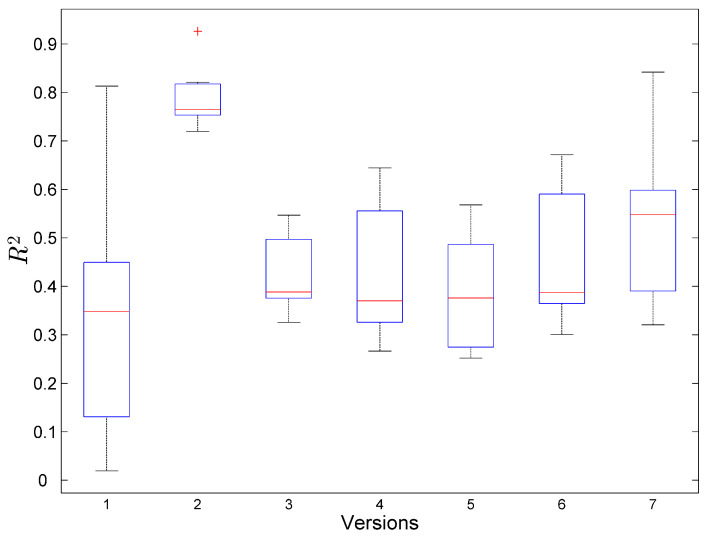
$R^{2}$ statistics for Leave-One-Out model validation.

**Figure 5:**
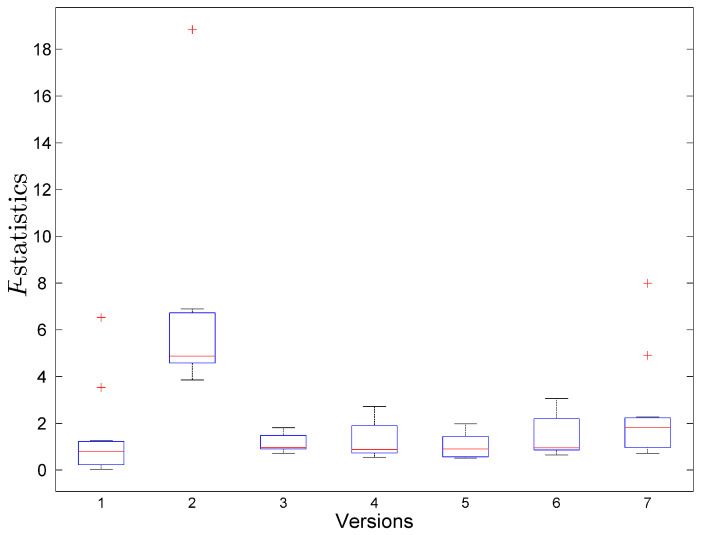
*F*-statistics for Leave-One-Out model validation.

Next, we have performed the outlier analysis and fit the model described by Equation (4) using Leave-One-Out validation, i.e., for each data column vector in Table 5, we use six values of data for model fitting, and the left out value is used for model evaluation. The process is repeated seven times, so that seven models are created. To evaluate the models, we use $R^{2}$ and *F*-statistics. The results are presented in Figure 4 and Figure 5. Both figures show the data for software version ver2 as an outlier, therefore, we remove its data from further analysis.

**Figure 6:**
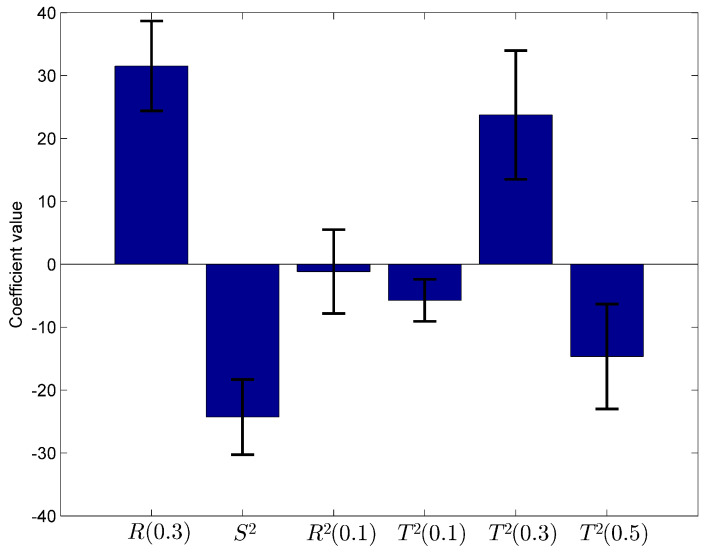
Coefficients of nonlinear regression model.

After calculating the regression coefficients using SPSS and Shannon, Rényi and Tsallis entropies, a model has been proposed and predict the bad smells which are likely to occur in the future. The best fitting model is described in Table 6 and Figure 6. The standard deviation values have been obtained using bootstrapping with 10,000 bootstrap samples.

## 6. Result and Discussion

### 6.1. Result

**Figure 7:**
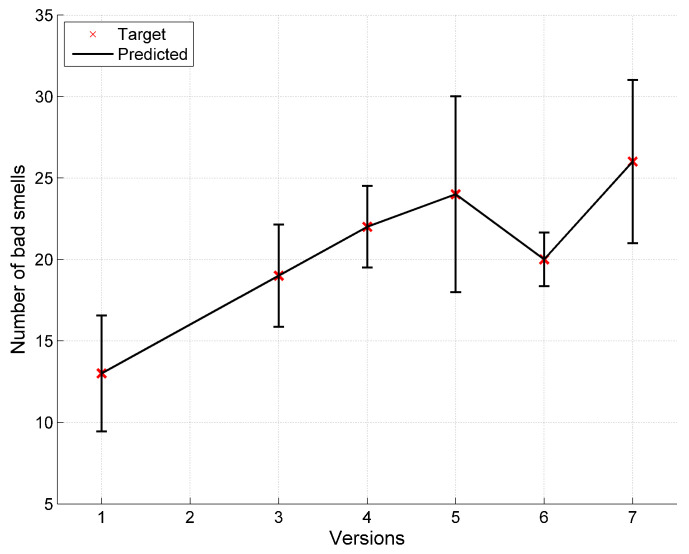
Target and model prediction values.

The data has been collected for seven versions of Apache Abdera. The entropy of the observed bad smells is estimated accordingly for different versions and the corresponding classes. The parameters for the bad smell prediction model have been calculated by applying the nonlinear regression using SPSS. Table 7 shows the predicted bad smells using the proposed model. In this table $O_{b}$ represents the observed values of the bad smells in the Apache Abdera project and *S* represents the predicted values of bad smells using Shannon entropy and *R*(0.1) and *T*(0.1) represents the predicted values of bad smells using Rényi and Tsallis entropies respectively with the α value starting from 0.1 upto 0.9. Table 5 contains the predicted bad smells as a result of proposed model in the Section 6.1. The modeling results are presented in Figure 7.

**Figure 8:**
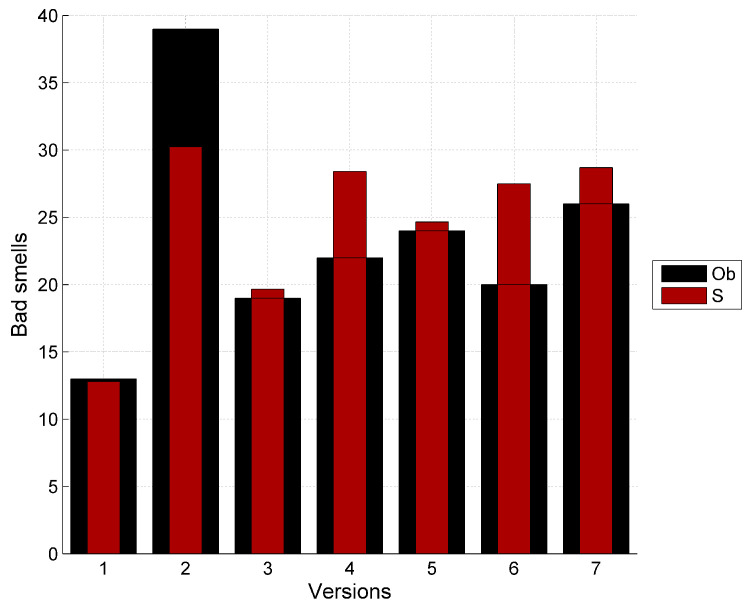
Graph between observed bad smells and predicted bad smells using Shannon entropy. $O_{b}$ represents the observed values of the bad smells in the Apache Abdera project; *S* represents the predicted values of bad smells using Shannon entropy.

**Figure 9:**
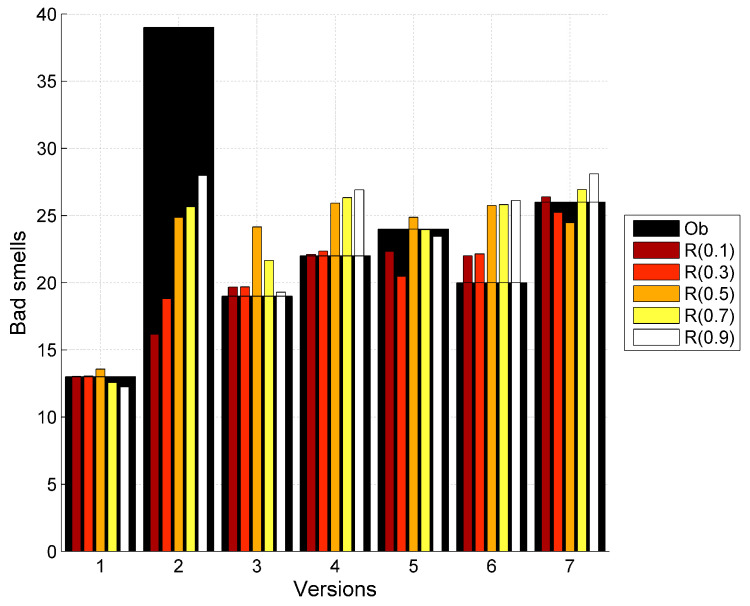
Graph between observed bad smells and predicted bad smells using Rényi entropy.

**Figure 10:**
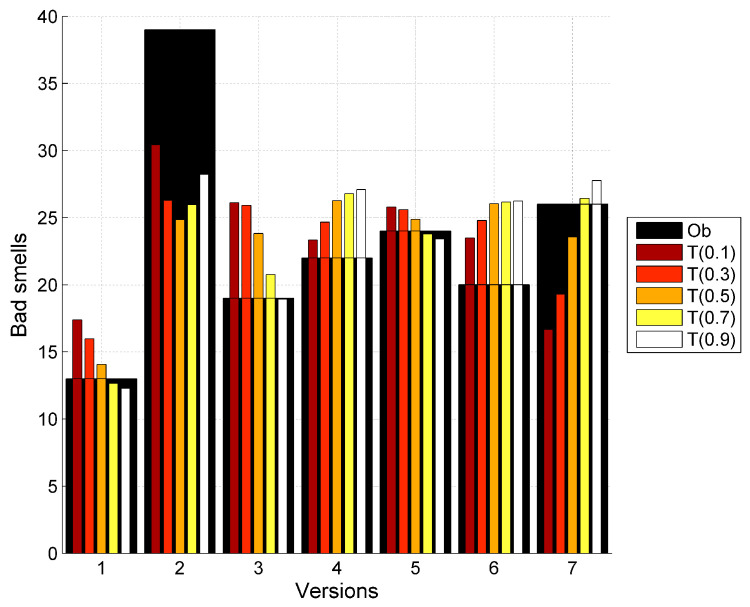
Graph between observed bad smells and predicted bad smells using Tsallis entropy.

Graphs showing the relationship between the observed bad smells and predicted bad smells using the proposed model with Shannon, Rényi and Tsallis entropies is shown in Figure 8, Figure 9 and Figure 10 respectively. Here Ob represents the observed bad smells. Y(S) represents the predicted bad smells with the help of Shannon’s entropy. Y(R(0.1)) represents the predicted bad smells with the help of Rényi’s entropy considering the value of α as 0.1 and further values of α as 0.3, 0.5, 0.7 and 0.9 are being considered. Y(T(0.1)) represents the predicted bad smells with the help of the Tsallis entropy and further values of α, similar to those used with Rényi’s entropy, are being considered. It is clear from these figures that the entropy and the bad smells are highly correlated with each other.

**Figure 11:**
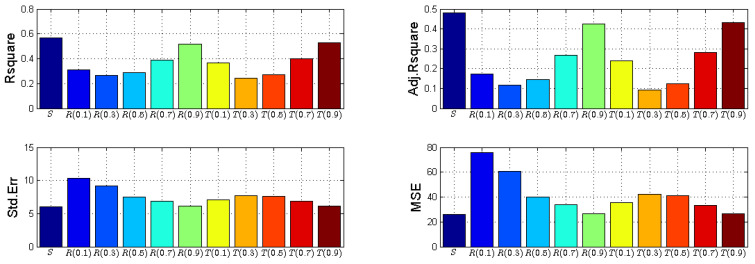
Statistical performance of entropies versus bad smells. Adj.: Adjusted; Std. Err: Standard Error; MSE: Mean Square Error.

The statistical performance parameters for the considered data sets are shown in Table 8 and Figure 11. We can conclude from Table 7 that $R^{2}$ and Adjusted $R^{2}$ is maximum that is 0.567 and 0.480 respectively for Shannon entropy. For Rényi and Tsallis entropy, it is observed that on increasing the α value from 0.1 to 0.9, the $R^{2}$ increases from 0.312 to 0.52 and from 0.367 to 0.527 and Adjusted $R^{2}$ also increases from 0.174 to 0.424 and from 0.240 to 0.432 respectively.

(5) $$\begin{array}{c} R^{2}=1-CorrectedSS/ResidualSS \end{array}$$

$R^{2}$ (or coefficient of determination) is the ratio of the sum of squares (SS) between the corrected and residual subtracted from 1. It is used to evaluate the level of significance between the predicted and observed values. $R^{2}$ estimates the total variation about the mean for the fitted curve. Its value ranges from 0 to 1. Higher value of $R^{2}$ indicates that the model fits the data well. It is also able to explain the variation in the data.

Adjusted $R^{2}$ explains the variation in the dependent variable. So, if the $R^{2}$ value is 0.567, it means that 56.7% of variance in dependent variable is predictable from the independent variable. Other statistical performance parameters are Standard Error (SE) and Mean Square Error (MSE). SE refers to an estimate of standard deviation to compute the estimate which is derived from a particular sample, and MSE refers to the average of the squared errors between actual and estimated data.

**Figure 12:**
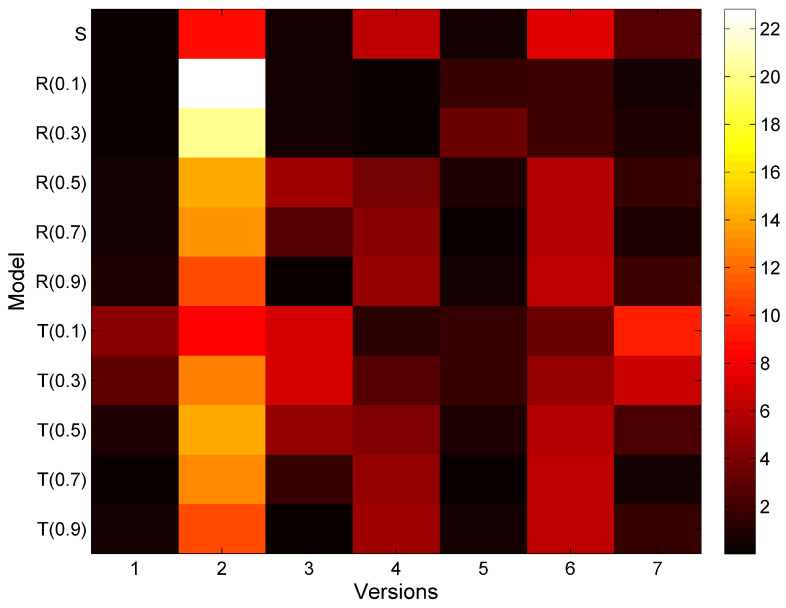
Comparison of models using goodness of fit parameter Prediction Error.

**Figure 13:**
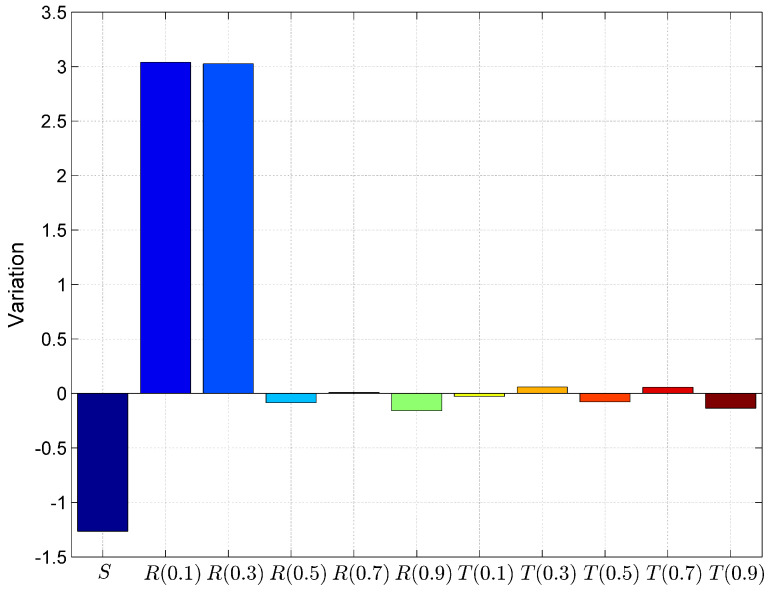
Comparison of models using goodness of fit parameter Bias.

**Figure 14:**
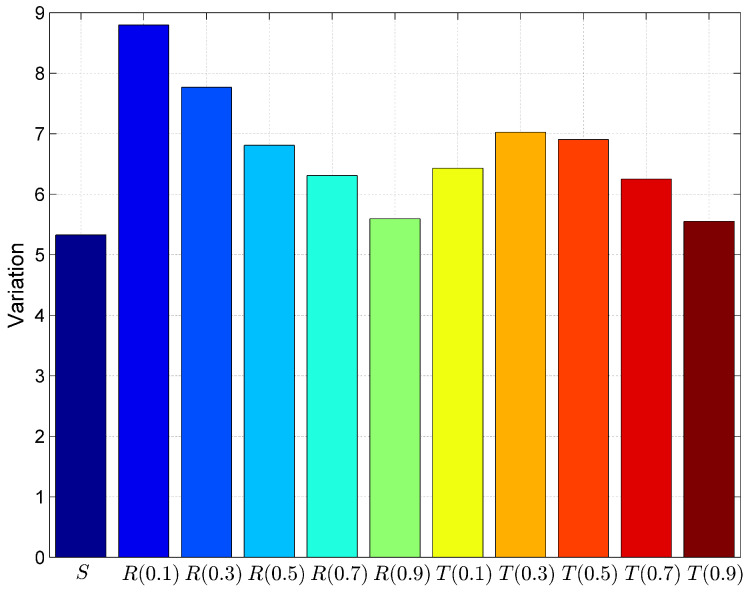
Comparison of models using goodness of fit parameter Variation.

**Figure 15:**
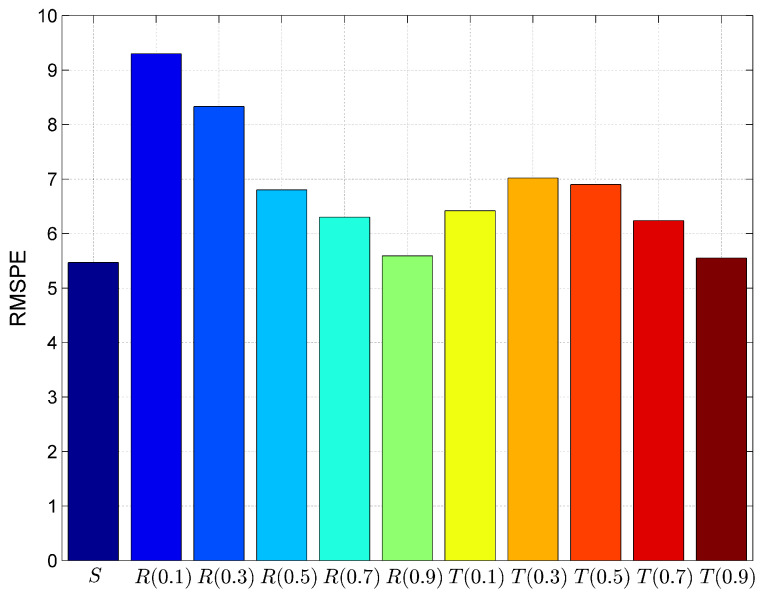
Comparison of models using goodness of fit parameter Root Mean Squared Prediction Error (RMSPE).

Further, we have calculated the goodness of fit parameters to validate the model, whether this statistical model meets the objectives. These parameters are prediction error, bias, variation and Root Mean Squared Prediction Error (RMSPE) as shown in Figure 12, Figure 13, Figure 14 and Figure 15. Prediction Error (PE) is the difference between the observed value and predicted value. The lower value of this parameter indicates less fitting error and it proves the goodness of fit is better.

(6) $$\begin{array}{c} PE=|ObservedValue-PredictedValue| \end{array}$$

Bias is the average value of prediction error. Lower value of bias implies higher goodness of fit.

(7) $$\begin{array}{c} Bias=\sum (ObservedValue-PredictedValue)/n \end{array}$$

Variation is the standard deviation of the prediction error. Lower value of variation provides higher goodness of fit.

(8) $$\begin{array}{c} Variation=\sqrt{\sum ((PE-Bias)\times (PE-Bias))/n-1} \end{array}$$

Root Mean Squared Prediction Error (RMSPE) is a measure of closeness to estimates the observations for a model. Lower value of this parameter provides higher goodness of fit.

(9) $$\begin{array}{c} RMSPE=\sqrt{Bias\times Bias+Variation\times Variation} \end{array}$$

### 6.2. Discussion

Our results show that the prediction model for bad smells using entropy (an information theory approach) can demonstrate significant performance. All three entropy approaches (Shannon, Rényi, Tsallis) are not performing equally in predicting the bad smells. Low values of error parameters like prediction error, bias, variation and RMSPE indicate higher goodness of fit for the proposed model. The value of $R^{2}$ (ranges from 0 to 1) should be large enough to explain the variation in the data and prove the model best fitted. There is one outlier value in the data set as shown in Table 4. This value is in class ServiceUtil and Version 2 of Apache Abdera. After removing that data; the value of $R^{2}$ rises up to 0.93, i.e., the model has fitted 93% and can explain 93% variation in the data.

## 7. Application and Limitation

The proposed model can help in predicting the bad smells in the software. It also can help to fix bad smells before they create problems for software quality and reliability. Generally, in a software life cycle, bad smell detection is performed after the implementation stage, whereas early bad smell prediction can improve software quality. It is beneficial to predict and remove the bad smells as early as possible. Prediction of software bad smells can help in determining code quality. In open-source projects any developer can participate in the development process. However, it is the duty at the managerial side to evaluate the quality of the developed code by a particular developer. Thus, the developers, who are developing the code with less bad smells can be promoted by the manager. In this way, this research will also help in deciding the appraisal of the employee in the software programming industry.

The limitation of this research is that only a subset of all 22 bad smells introduced by Fowler et al. [17] is considered. We have collected data of all seven versions of open-source project Apache Abdera, which are available online. In this research, open-source software has been examined whereas closed source (proprietary) software can also be taken into consideration in the future. In addition, only three entropy approaches were used for model development.

### Threats to Validity

The main threat related to the software engineering experimentation work is the relationship between theory and observations [61] (construct validity). In the context of this research, code smell measurements are performed with the Robusta tool. We can not ignore that Robusta excludes some smells and there are also distinct threshold values of bad smells in other existing tools which may affect the bad smells observations. External validity are concerned with generalization of the results [61]. The model presented here supports only Java software. For generalization of results, the model needs to be implemented for other languages too. Hence, the replicated study is needed for generalization. Threats to the internal validity concern about the internal factors of our research experiment design [61]. Although a non-linear regression model is proposed for bad smell prediction yet different statistical methods can lead to another direction for predicting bad smells.

## 8. Conclusions

Software quality is highly affected by the bad smells in a project. Bad smells are introduced when there are changes in the software code, feature enhancement/modification or new features are introduced in the software. We have proposed a statistical model using an information theoretic approach with nonlinear regression for predicting the bad smells. Previous studies have discussed a number of detection techniques of bad smells, while we have made a first attempt to formulate a mathematical model for bad smell prediction. We validate the model using statistical tests. The experiment results have shown that all the three entropy approaches (Shannon, Rényi and Tsallis) are sufficient to predict the bad smells in software. We have validated the model on the basis of $R^{2}$ value, which is largest for Shannon’s entropy, i.e., $R^{2}=0.567$. When we remove one outlier value in the data set, the value of $R^{2}$ increases to 0.93. The predicted bad smells help in maintaining the quality of the software and in reducing the developing time as well.

This model can be applied in the future versions of the Apache Abdera software as well as other Java software using the methodology suggested in the paper. This will help software companies to predict bad smells in the earlier stage of the software life cycle. This research can be further extended with other measures of entropy with different parameter values. The study may be extended to another open-source project as well as closed source projects with different modules.

## Figures and Tables

**Table 1:** Comparison between the proposed and prior research works.

	Bad Smell Observations with Software Classes	Bad Smell Detection	Mathematical Model	Entropy Based Approach	Industrial Application
Proposed Approach	Yes	Yes	Yes	Yes	Yes
Tufano et al. [7,19]	No	Yes	No	No	Yes
Maneerat et al. [12]	No	Yes	No	No	No
Bansal [49]	No	No	No	No	No
Zhu et al. [47]	No	No	Yes	No	No
Amarjeet et al. [48]	No	No	No	No	No
Holschuh [44]	No	Yes	No	No	Yes
Czibula et al. [31]	Yes	Yes	No	No	No
Chaturvedi et al. [53]	No	No	Yes	Yes	No
Singh et al. [52]	No	No	Yes	Yes	No

**Table 2:** Example showing the number of bad smells in classes with respect to versions.

Class	Version 1	Version 2	Version 3
C1	*	*	*
C2	*	-	*
C3	**	**	-
C4	*	**	*

Note: *: 1 smell, **: 2 smells.

**Table 3:** Description of code smells.

s. no	Name	Description
1	Empty Catch Block	When the catch block is left blank in the catch statement.
2	Dummy Handler	Dummy handler is only used for viewing the exception but it will not handle the exception.
3	Nested Try Statements	When one or more try statements are contained in the try statement.
4	Unprotected Main	Outer exception will not be handled in the main program; it can only be handled in a subprogram or a function.
5	Careless Cleanup	The exception resource can be interrupted by another exception.
6	Exception thrown in the finally block	How to handle the exception thrown inside the finally block of another try catch statement.

**Table 4:** Data of Detected Code Smells in the classes of Apache Abdera Software.

Class Name	Bad Smells In Each Version of the Software						
	ver1	ver2	ver3	ver4	ver5	ver6	ver7
CacheControlUtil	0	1	1	1	1	1	0
AbstractExtensionFactory	0	1	1	1	1	1	0
CompressionUtil	0	1	1	1	1	1	0
MimeTypeHelper	0	6	0	6	6	6	4
ServiceUtil	5	15	1	0	0	0	14
UrlEncoding	0	3	1	1	1	1	0
DHEnc	0	1	1	1	1	1	0
Security Base	0	1	4	4	4	0	0
URITemplates	0	1	1	1	1	1	0
ThreadHelper	0	2	2	2	2	2	2
FOMWriter	0	2	2	0	2	2	0
SimpleAdapter	0	1	1	1	1	1	0
BaseResponseContext	0	2	2	2	2	2	0
Test	6	1	0	0	0	0	0
XsltExample	1	0	0	0	0	0	2
AbderaResult	1	0	0	0	0	0	2
Enc	0	1	1	1	1	1	0
SimpleCache Key	0	0	0	0	0	0	1
Escaping	0	0	0	0	0	0	1
Total	13	39	19	22	24	20	26

**Table 5:** Different entropies for each version with different values of α.

*S*	R(0.1)	R(0.3)	R(0.5)	R(0.7)	R(0.9)	T(0.1)	T(0.3)	T(0.5)	T(0.7)	T(0.9)
1.61	1.95	1.86	1.78	1.71	1.64	2.65	2.10	1.71	1.42	1.21
3.11	3.84	3.7	3.54	3.37	3.2	11.09	7.16	4.82	3.38	2.48
3.51	3.68	3.65	3.61	3.57	3.53	9.95	6.96	4.99	3.67	2.77
3.20	3.55	3.48	3.41	3.33	3.25	9.08	6.31	4.51	3.33	2.53
3.35	3.54	3.61	3.54	3.46	3.39	9.85	6.78	4.81	3.519	2.65
3.24	3.55	3.49	3.43	3.36	3.28	9.11	6.36	4.57	3.37	2.56
2.11	2.74	2.60	2.46	2.32	2.18	5.038	3.63	2.70	2.069	1.63

**Table 6:** Nonlinear regression model.

Dependent Variable	Coefficient	Std. Deviation of Coefficient
*R*(0.3)	31.534	7.268
$S^{2}$	−24.284	6.169
$R^{2}$(0.1)	−1.155	6.83
$T^{2}$(0.1)	−5.728	3.323
$T^{2}$(0.3)	23.751	10.283
$T^{2}$(0.5)	−14.649	8.498

**Table 7:** Predicted bad smells (*Y*) using proposed model.

Ob	*S*	R(0.1)	R(0.3)	R(0.5)	R(0.7)	R(0.9)	T(0.1)	T(0.3)	T(0.5)	T(0.7)	T(0.9)
13	12.81	13.02	13.06	13.57	12.57	12.23	17.39	15.99	14.07	12.66	12.27
39	30.24	16.16	18.81	24.86	25.65	28.0	30.42	26.3	24.86	25.97	28.23
19	19.66	19.68	19.70	24.15	21.67	19.30	26.12	25.92	23.82	20.77	18.93
22	28.4	22.11	22.37	25.92	26.33	26.92	23.35	24.67	26.27	26.8	27.10
24	24.65	22.33	20.47	24.88	23.94	23.44	25.79	25.59	24.89	23.79	23.42
20	27.49	22.01	22.15	25.74	25.82	26.11	23.50	24.79	26.05	26.17	26.25
26	28.69	26.39	25.23	24.47	26.94	28.10	16.66	19.3	23.57	26.44	27.77

**Table 8:** Statistical performance of entropies vs bad smells.

Entropy	Parameter (α)	Rsquare	Adj. Rsquare	Std. Err	MSE
Shannon	-	0.567	0.4804	6.0261	25.939
Rényi	0.1	0.312	0.1744	10.2862	75.5764
	0.3	0.264	0.1168	9.2305	60.8594
	0.5	0.289	0.1468	7.4598	39.7493
	0.7	0.39	0.268	6.9112	34.1185
	0.9	0.52	0.424	6.131	26.8495
Tsallis	0.1	0.367	0.2404	7.0405	35.4062
	0.3	0.244	0.0928	7.693	42.2734
	0.5	0.27	0.124	7.5623	40.8495
	0.7	0.4	0.28	6.8458	33.4757
	0.9	0.527	0.4324	6.0847	26.4462

Adj.: Adjusted; Std. Err: Standard Error; MSE: Mean Square Error.
